# 4‐*O*‐Methylglucuronoxylan from *Hygrophila Ringens* var. *Ringens* Seeds: Chemical Composition and Anti‐Inflammatory Activity

**DOI:** 10.1002/mabi.202400434

**Published:** 2025-01-13

**Authors:** Vo Hoai Bac, Tat Cuong Trinh, Andreas Koschella, Thomas Heinze, Yu Ping Fu, Kari Tvete Inngjerdingen, Le Van Truong, Berit Smestad Paulsen, Martin Gericke

**Affiliations:** ^1^ Institute of Biotechnology Vietnam Academy of Science and Technology 18 Hoang Quoc Viet Hanoi 100 000 Vietnam; ^2^ Graduate University of Science and Technology Vietnam Academy of Science and Technology 18 Hoang Quoc Viet Hanoi 100 000 Vietnam; ^3^ Key Laboratory for Enzyme and Protein Technology Hanoi University of Science Hanoi 100 000 Vietnam; ^4^ Friedrich Schiller University Jena Institute for Organic Chemistry and Macromolecular Chemistry Center of Excellence for Polysaccharide Research Humboldtstraße 10 D‐07743 Jena Germany; ^5^ Department of Pharmacy Section for Pharmaceutical Chemistry University of Oslo Oslo 0316 Norway

**Keywords:** 4‐O‐methylglucuronoxylan, anti‐inflammatory, cytokine, *Hygrophila ringens* var. *ringens*, isolation, polysaccharide

## Abstract

*Hygrophila ringens* var. *ringens* is a medicinal plant of the Acanthaceae family. A soluble polysaccharide is extracted from *H. ringens* seeds using warm water, followed by deproteinization and purification using column chromatography. DL1 is characterized comprehensively using spectroscopic and chromatographic techniques and identified as a polymer containing xylose (Xyl; 78.5%) and 4‐*O*‐methyl‐*d*‐glucuronic acid (4‐*O*‐MeGlcA; 21.5 %). The most prominent glycosidic linkages detected are terminal‐xylose (T‐Xyl); 1,2,3,4‐Xyl*p*; 1,2,4‐Xyl*p*; and T‐4‐*O*‐MeGlcA. DL1 belongs to the xylan group and is a 4‐*O*‐methylglucuronoxylan. DL1 exhibits inhibition of bovine serum albumin denaturation with IC_50_ values of 0.35 mg mL^−1^ and a similar activity to diclofenac (non‐steroidal anti‐inflammatory drug). In a model of lipopolysaccharide‐stimulated macrophages, DL1 (20–40 µg mL^−1^) strongly inhibits inflammatory cytokines and reactive oxygen species release without having significant macrophage cytotoxicity. The inhibitory effect of DL1 on inflammatory cytokines is mediated by the activation of mitogen‐activated protein kinases by inhibiting the phosphorylation of p38 and extracellular signal‐regulated kinase. These results highlight the potential of DL1 for treating inflammation through its cytokine‐suppressive activity.

## Graphical abstract

4‐*O*‐Methylglucuronoxylan from *Hygrophila ringens* var. *ringens* is found to inhibit the serum albumin denaturation, inflammatory cytokines, and release of reactive oxygen species by activation of mitogen‐activated protein kinases without having significant macrophage cytotoxicity. Inhibition is mediated by the activation of mitogen‐activated protein kinases by inhibiting the phosphorylation of p38 and extracellular signal‐regulated kinase.

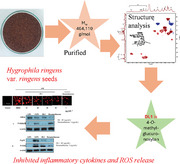

## 1 Introduction

In recent years, polysaccharides from natural sources such as β‐glucans,^[^ ^1^ ^]^ pectins,^[^ ^2^ ^]^ galactomannans,^[^ ^3^ ^]^ and xylans,^[^ ^4^ ^]^ have attracted interest owing to their important anti‐inflammatory and immunomodulatory effects.^[^ ^4^ ^]^ Previous studies have demonstrated that xylans and heteroxylans exhibit multiple bioactivities. For example, a heteroxylan from *Maytenus ilicifolia* (*M. ilicifolia*) leaves has been reported to exhibit anti‐ulcer activity,^[^ ^5^ ^]^ while arabinoxylan fractions from tomato seed mucilage have antinociceptive activities.^[^ ^6^ ^]^ A glucuronoxylan from the roots of *Cudrania tricuspidate* (*C. tricuspidate*) has been reported to exhibit immunomodulatory activity.^[^ ^7^ ^]^ Furthermore, a 4‐*O*‐methylglucuronoxylan isolated from *Castanea sativa* (*C. sativa*) extracts has been shown to inhibit human epidermoid carcinoma cells,^[^ ^8^ ^]^ while 4‐*O*‐methylglucuronoxylan isolated from *Aralia echinocaulis* (*A. echinocaulis*) exhibits inhibitory activity against colon cancer cells.^[^ ^9^ ^]^

Plants of the Acanthaceae family play a key role in the treatment of many diseases and have been extensively studied pharmacognostically.^[^ ^10^ ^]^ Previous studies have identified a polysaccharide in the medicinal plant *Acanthus ebracteatus* (*A. ebracteatus*) Vahl (family Acanthaceae) that contains a special sugar, 3‐*O*‐methylgalactose. This polysaccharide affects the complement of the immune system.^[^ ^11^ ^]^ *Pseuderanthemum carruthersii* (*P. carruthersii;* family Acanthaceae) also contains a neutral polysaccharide composed of 3‐*O*‐methylgalactose, exhibiting anti‐inflammatory effects.^[^ ^12^ ^]^ Synthetic drugs used for anti‐inflammatory therapy are often associated with toxicity and adverse effects. Currently, plant‐derived medicines are used as alternatives or complementary medications to glucocorticoids and non‐steroidal anti‐inflammatory agents. They exhibit lower toxicity and, therefore, may be used for the long‐term treatment of chronic inflammation.^[^ ^13^ ^]^

*Hygrophila ringens* var. *ringens* belongs to the Acanthaceae family. The Vietnamese common names are Đình Lịch, Bình Lịch, Mịch Lịch, Huỳnh Lịch, and Ngũ Sắc. This species grows in the wild along the hillsides of Vietnam and on bare land, coastal ditches, and wastelands. *H. ringens* is an herbaceous plant that is up to 1 m in height. The flowering season is from May to December. In traditional Vietnamese medicine, the leaves of *H. ringens* are used to treat wounds, swelling, pharyngitis, and mastitis. The seeds are used to treat headaches, fever, and skin inflammation.^[^ ^14^ ^]^ Dinh Lich seeds are as small as sesame seeds and light brown in color. Their outer part is decorated with hairs that absorb water. When soaked in hot water, a viscous layer forms around the seeds, which causes the seeds to combine with each other. Seeds soaked in water form a slimy mass that can treat pustules, acne, and skin inflammation. The *H. ringens* seeds are also used to treat painful wounded skin. To date, no studies have been conducted on the components and biological effects of this medicinal plant.

Macrophages are a part of the first line of defense in the body. During the inflammatory response, macrophages produce inflammatory mediators, nitric oxide, and cytokines (tumor necrosis factor, TNF, interleukin, IL‐1, IL6, and IL‐10). In systemic inflammatory response syndromes, macrophages release potent inflammatory cytokines that play important roles in pathological processes. A low amount of TNF‐α may contribute to host defense by limiting the spread of pathogens. However, an excessive increase in the number of TNF‐α in the inflammatory response leads to chronic inflammation, autoimmune diseases, tissue damage, and heart failure.^[^ ^15^ ^]^ Many studies have shown that persistently high levels of IL‐6 in the blood of patients with severe infections lead to a decline in the function of the blood microcirculation system and organs in the body. Excessive increases in IL‐6 levels are associated with chronic inflammatory diseases and cytokine storms caused by the coronavirus disease.^[^ ^16^ ^]^ IL‐8 is a cytokine that stimulates and disrupts the function of neutrophils, leading to excessive release of inflammatory molecules, damage to lung tissue, and neonatal sepsis.^[^ ^17^ ^]^ Thus, controlling cytokine production may reduce the likelihood of excessive inflammation in patients with severe infections.

The objective of this study was to characterize the structure of polysaccharides isolated from *H. ringens* seeds and evaluate their anti‐inflammatory activities in macrophages. In other words, we aimed to answer the following questions: What is the molecular structure of the isolated polysaccharides? Do the polysaccharides exhibit anti‐inflammatory activities? Therefore, in this study, we aimed to unravel the role of polysaccharides from *H. ringens* seeds on macrophages during inflammatory responses.

## 2 Results and Discussion

### 2.1 Polysaccharide Isolation

**Figure 1:**
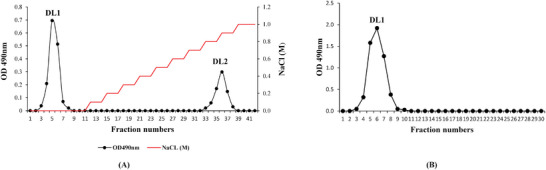
A) Elution profiles of the polysaccharide extract from *Hygrophila ringens* var. *ringens* seeds on a diethylaminoethyl (DEAE)‐cellulose 52 column. B) DL1 (peak 1 of part A) analyzed on a Sephadex G‐100. (A). The polysaccharide was eluted with distilled water followed by elution with a NaCl solution (0–1.0 m; 1 mL min^−1^). Fractions of 5 mL were collected (Optical density (OD) at 490 nm). B) The column was eluted with water at a flow rate of 0.5 mL min^−1^. Fractions of 5 mL were collected (Optical density, OD, at 490 nm).

Polysaccharides were extracted from *H*. *ringens* seeds using warm water, followed by precipitation with ethanol. A polysaccharide yield of 12.3 ± 2.1% (dry material) was obtained. The soluble polysaccharides were loaded onto a diethylaminoethyl (DEAE)–cellulose column. Two peaks were observed in the elution profile (**Figure** **1**A); fraction 1 was eluted with distilled H_2_O and fraction 2 was eluted with 0.8–0.9 m NaCl. Fraction 1 contained the main part of the polysaccharides and was therefore collected as sample DL1, which was used in further studies to determine its biological activity. The polysaccharide content in peak 2 (DL2) was very low (data not shown). DL1 was subjected to Sephadex G‐100 column chromatography and was found to be uniform (Figure 1B). The purified polysaccharide was examined using UV–Vis spectrophotometry in the wavelength range of 200–700 nm to confirm the absence of proteins and other non‐polysaccharide compounds.^[^ ^18^ ^]^

**Figure 2:**
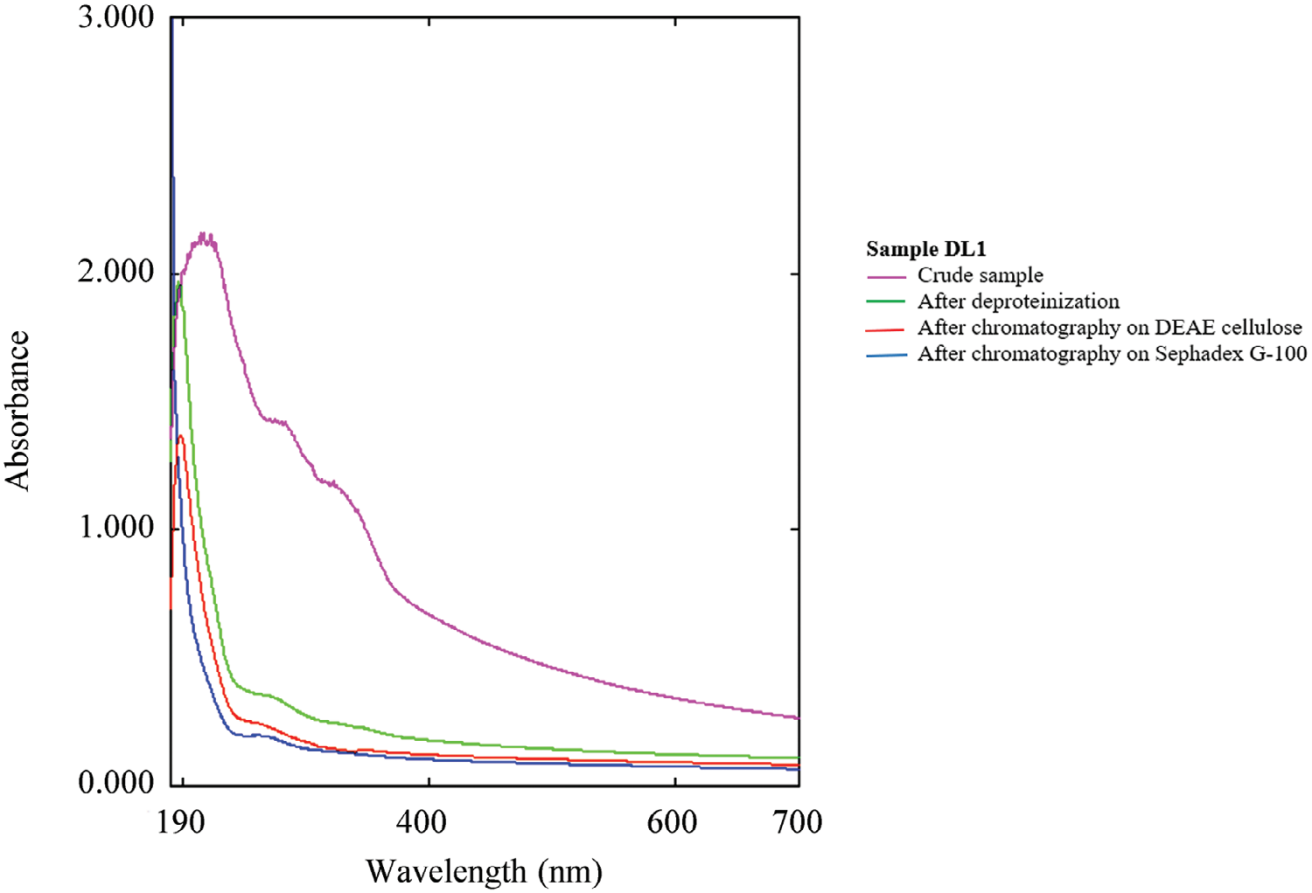
The UV‐Vis absorbance spectra of polysaccharide DL1 from seeds of *Hygrophila ringens* var. *ringens* (trichloroacetic acid, TCA; diethylaminoethyl, DEAE).

UV–Vis spectroscopy (**Figure** **2**) revealed that the purified polysaccharides only exhibited an absorption peak at 210 nm, which is the characteristic UV absorption peak of a polysaccharide.

**Figure 3:**
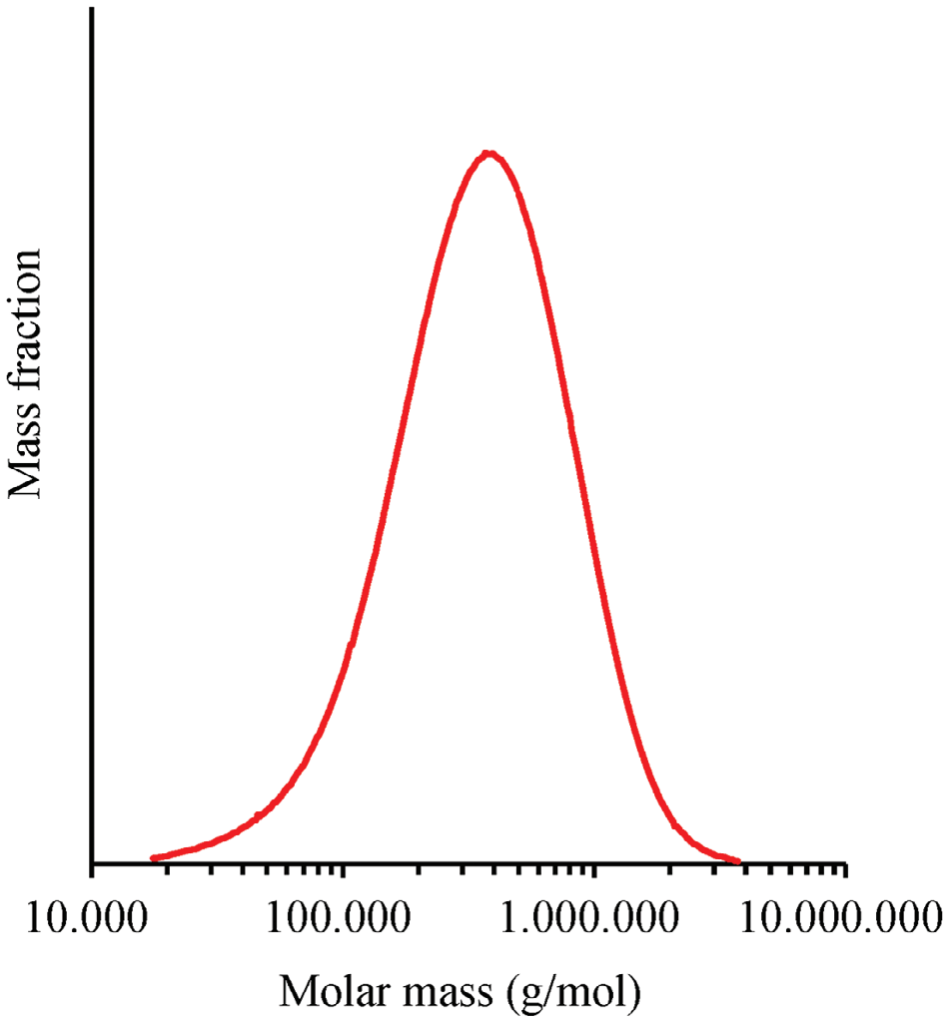
Molecular mass distribution of DL1 determined via size exclusion chromatography.

The absorbance of nucleic acids and proteins at 260 and 280 nm, respectively, could not be detected. The absence of proteins in DL1 was confirmed using the Lowry method^[^ ^19^ ^]^ (data not shown). The size‐exclusion chromatography (SEC) of DL1 revealed a monomodal molar mass distribution with a symmetrically sharp curve (**Figure** **3**). A weight‐averaged molecular weight of 464,110 g mol^−1^ was calculated.

### 2.2 Monosaccharide Composition, Linkage, and Structural Characterization

The results of monosaccharide composition analysis of DL1 are summarized in **Table** **1** and Figure S1 (Supporting Information). This purified polysaccharide is a water‐soluble polymer rich in xylose (Xyl; 78.5%) containing 4‐*O*‐methylglucuronic acid (4‐*O*‐MeGlcA; 21.5%) and traces of arabinose, rhamnose, fucose, mannose, galactose, glucose, galacturonic acid, and glucuronic acid.

**Table 1:** Monosaccharide composition (mol%, of total carbohydrate content) of DL1 from *Hygrophila ringens* var. *ringens* seeds after methanolysis.

Monosaccharide	mol%
Xylose	78.5
4‐*O*‐methyl‐*d*‐glucuronic acid	21.5

The results indicated that the isolated polysaccharide DL1 was a new 4‐*O*‐methylglucuronoxylan mainly composed of xylan and 4‐*O*‐MeGlcA. In a previous study, a 4‐*O*‐methylglucuronoxylan was isolated from *C. sati*, containing Xyl and 4‐*O*‐MeGlcA in a ratio of 5.9:1.^[^ ^20^ ^]^ The ratio of Xyl/4‐*O*‐MeGlcA in 4‐*O*‐methyl glucuronoxylan depends on the xylan source, and ratios ranging from 4:1 to 16:1 have been reported,^[^ ^21^ ^]^ with *M. ilicifolia* leaves containing an acidic heteroxylan with a high xylose content (76%).

**Table** **2** and Figures S2 and S3 (Supporting Information) show the most prominent glycosidic linkages in DL1: T‐Xyl; 1,2,4‐Xyl*p*; 1,2,3,4‐Xyl*p*; and T‐4‐*O*‐Me‐Glc*p*A.

**Table 2:** Glycosidic linkage types (mol%) of the xylose (Xyl)‐ and 4‐*O*‐methylglucuronic acid (4‐*O*‐MeGlcA)‐repeating units present in DL1.

Linkage types		Rt min^−1^	Primary fragments	DL1
Xyl*p*	T‐	13.33	117, 118, 161, 162	33.2
	1,3,4‐	17.83	118, 261	5.6
	1,2,4‐	17.91	189,190	18.8
	1,2,3,4‐	19.61	145, 146, 217, 218, 290	20.9
4‐*O*‐Me‐Glc*p*A	T‐	16.63	47, 118, 162, 163, 207	21.5

**Figure 4:**
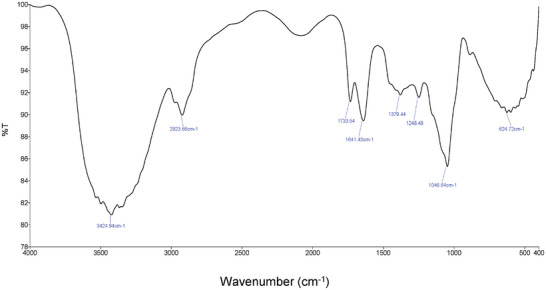
FTIR spectrum of DL1.

The FTIR spectrum of DL1 exhibited a distinct stretching vibration of hydroxy groups at 3425 cm^−1^ and absorption bands at 2924 cm^−1^ attributed to CH stretching vibrations (**Figure** **4**). These peaks were assigned to the characteristic absorption of polysaccharides and were similar to those of glucuronoxylans from *Dolichos lablab* (*D. lablab*) L. hull.^[^ ^22^ ^]^

**Figure 5:**
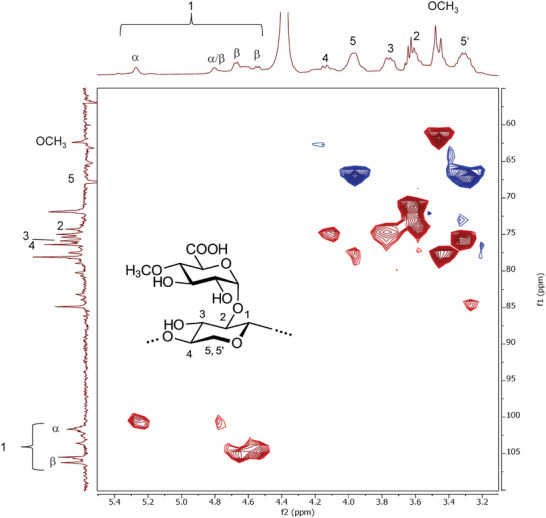
HSQC/DEPT NMR spectrum of DL1.

The peak at 1643 cm^−1^ results from absorbed water.^[^ ^23^ ^]^ The presence of *O*‐acetyl groups was demonstrated by the band at ≈1248 cm^−1^.^[^ ^24^ ^]^ The vibration between 1200 and 1000 cm^−1^ was assigned to stretching vibrations of the OH groups and the C─O─C of the glycosidic bond.^[^ ^22^ ^]^ The peak between 1200 and 1000 cm^−1^ indicated that the sample mainly consisted of 4‐*O*‐methylglucuronoxylan.^[^ ^25^ ^]^ The molecular structure of polysaccharide DL1 was also analyzed via NMR spectroscopy (**Figures** **5** and S4, Supporting Information).

The ^1^H‐NMR spectrum of DL1 exhibited characteristic polysaccharide signals in the range of 3.5–5.5 ppm (Figure S5, Supporting Information). NMR analysis of DL1 revealed its complex structure, exhibiting typical resonances of the carbonyl carbon atoms at 178.4, 175.5, and 173.8 ppm, indicating that the uronic acids are esterified. These findings are in accordance with the uronic acid content. Moreover, the small peak at 23.3 ppm is attributed to a methyl group. Thus, the presence of acetyl moieties was confirmed. Several peaks between 5.4 and 4.5 ppm appear in the ^1^H‐NMR spectrum that could be assigned as anomeric protons (H1) having both α‐ (5.7–5.5 ppm) and β‐configuration (4.7–4.5 ppm). The resonances of the main chain can be assigned as follows: 4.67/105.6 ppm (H1/C1), 3.59/71.9 ppm (H2/C2), 3.78/75.0 ppm (H3/C3), 4.11/75.1 ppm (H4/C4), as well as 3.97/67.8 and 3.37/67.8 ppm (H5/C5). Position 5 exhibits diastereotopic signal splitting owing to the neighboring position 4. Thus, the polymer consisted largely of xylose. Notably, there was a significantly higher content of 1,2,4‐linked Xyl*p* (18.8%). This hypothesis is underpinned by the amount of terminal Xyl*p* detected (33.2%). Therefore, the polysaccharide DL1 is highly branched or has different Xyl*p* units within the polymer. Based on their correlations in the HSQC/DEPT‐NMR spectrum (Figure 5), these Xyl*p* residues may possess both α‐ and β‐configurations. Moreover, the significant amount of 4‐*O*‐MeGlcA (21.5%) causes resonances at 3.4 ppm in the ^1^H‐NMR and 60.0 ppm in the ^13^C‐NMR spectra, which are attributed to the methoxy group (─OCH_3_) of 4‐*O*‐MeGlcA. Consequently, DL1 can be classified as 4‐*O*‐methylglucuronoxylan. Further conclusions on the molecular structure based on NMR experiments cannot be drawn because of the lack of a high‐frequency spectrometer to boost the resolution. As shown in the HSQC‐DEPT‐NMR spectrum, at least 10 different C/H‐couplings were attributed to the anomeric position. They were almost equally distributed between the α‐ and β‐anomers.

### 2.3 In Vitro Inhibition of Bovine Serum Albumin Denaturation

The thermal inhibition of bovine serum albumin (BSA) denaturation is an *in vitro* procedure used to screen plant compounds for anti‐inflammatory activity.^[^ ^26^ ^]^ Diclofenac is a non‐steroidal anti‐inflammatory drug (NSAID) that has stronger anti‐inflammatory activity than other NSAIDs^[^ ^27^ ^]^ and was therefore used as the positive control.

**Figure 6:**
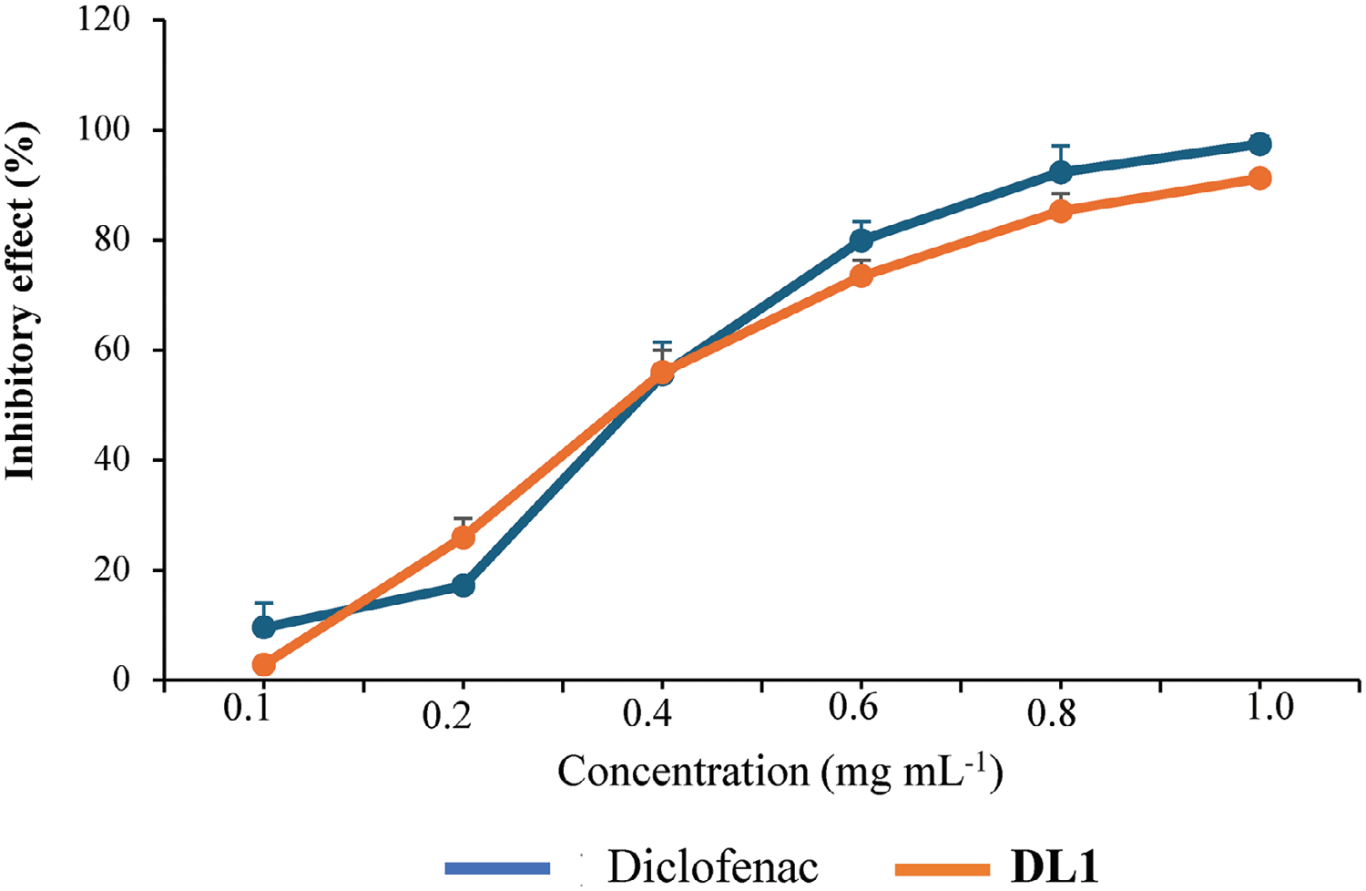
The effects of diclofenac and DL1 on bovine serum albumin thermal denaturation.

When administered at concentrations between 0.1—1 mg mL^−1^, DL1 showed a concentration‐dependent reduction in albumin denaturation. Therefore, DL1 prevents bovine serum albumin denaturation with IC_50_ values of 0.35 mg mL^−1^ and has similar activity to diclofenac (**Figures** **6** and S5, Supporting Information). This inhibitory effect increased with increasing concentration. At a concentration of 1 mg mL^−1^, an inhibitory effect of 97.45 ± 1.49% was observed for diclofenac and 91.23 ± 0.46% for DL1. Synthetic chemical medicines (diclofenac) have potent anti‐inflammatory properties; however, their prolonged use may cause complications and side effects in humans. DL1 from *H*. *ringens* seeds effectively inhibited BSA degradation (IC_50 _= 0.35 mg mL^−1^) at a lower dose compared to the galactomannans extracted from *Alhagi maurorum* (*A. maurorum*) Medik seeds (IC_50 _= 5.8 mg mL^−1^).^[^ ^28^ ^]^

### 2.4 Cytotoxicity and Anti‐Inflammatory Effect of DL1 on Macrophage

The cytotoxicity of DL1 was tested against RAW 264.7 cells. DL1 exhibited no cytotoxicity on RAW 264.7 cells at a concentration range of 10–40 µg mL^−1^ (Figure S6, Supporting Information). Thus, a concentration of DL1 between 10 and 40 µg mL^−1^ was used for further experiments.

**Figure 7:**
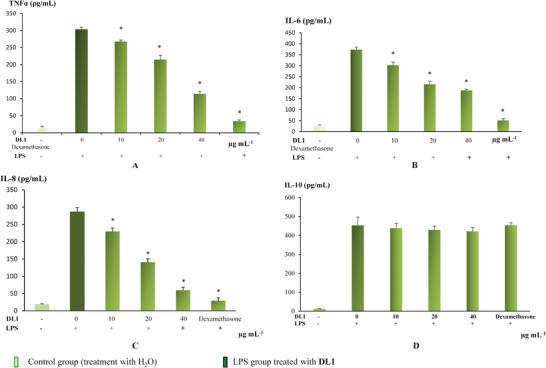
Effect of DL1 on LPS‐induced proinflammatory cytokines production TNFα (A), IL‐6 (B), IL‐8 (C), and IL10 (D). *p < 0.05 compared to LPS group.

LPS‐induced RAW 264.7 macrophage is an in vitro model for screening the anti‐inflammatory activities of compounds.^[^ ^29^ ^]^ **Figure** **7** shows that all the LPS‐induced macrophages released high levels of the cytokines TNFα, IL‐6, IL‐8, and IL‐10. In systemic inflammatory response syndromes, macrophages release potent inflammatory cytokines that play important roles in pathological processes.

The TNF‐α concentration decreased significantly immediately after treatment with DL1 (10 µg mL^−1^, Figure 7A). At a DL1 concentration of 40 µg mL^−1^, TNF‐α decreased by 62.4% compared to the positive control (*p* < 0.05). DL1 strongly inhibited IL‐6 release; at the highest concentration (40 µg mL^−1^), DL1 inhibited IL‐6 by ≈49.6% compared to the positive control (*p* < 0.05, Figure 7B). In particular, DL1 (40 µg mL^−1^) strongly inhibited IL‐8 by ≈80%, which is almost equal to dexamethasone treatment (an anti‐inflammatory corticosteroid drug, Figure 7C). Furthermore, DL1 did not influence IL‐10 release (Figure 7D). Several studies have shown that plant polysaccharides are a potential medicinal source to replace glucocorticoid anti‐inflammatory drugs. Acemannan, a polysaccharide from Aloe vera, has anti‐inflammatory properties and has been used in anti‐acne cosmetic products to reduce inflammation.^[^ ^30^ ^]^ Arabinoxylan fractions from tomato seed mucilage exhibited anti‐inflammatory properties,^[^ ^31^ ^]^ while xylan from red seaweeds showed anti‐inflammatory effects and improved scratch wound healing.^[^ ^32^ ^]^ 4‐*O*‐methylglucuronoxylan from *A. echinocaulis* potently inhibits colon cancer cells,^[^ ^13^ ^]^ while glucuronoxylan (CTPB1) from the roots of *C. tricuspidate* is composed of xylose and 4‐*O*‐methyl*‐d*‐glucuronic acid, which have immunomodulatory activities.^[^ ^11^ ^]^ The inhibitory effect of DL1 on inflammatory cytokines was strong and similar to that of polysaccharides from *P. carruthersii*, a medicinal plant belonging to the *Acanthaceae* family.^[^ ^3^ ^]^ Therefore, DL1 has a promising potential for use as an anti‐inflammatory drug.

**Figure 8:**
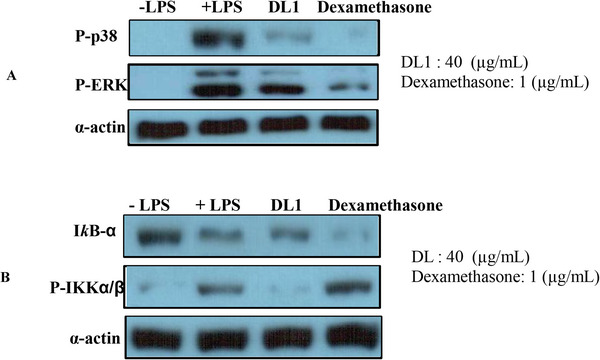
Effects of DL1 on the signaling pathways of RAW264.7 cells by Western blot assay. MAPKs (A) and NF‐κB (B).

The anti‐inflammatory activity of polysaccharides from medicinal plants is based on the inhibition of activation of the NF‐κB and the mitogen‐activated protein kinase (MAPK) signaling pathways in macrophages.^[^ ^33^ ^]^ DL1 (40 µg mL^−1^) suppressed the phosphorylation of p38 (P‐p38) and P‐ERK compared to the LPS group (**Figure** 8 part A). Our results indicated that the inhibitory effect of DL1 on inflammatory cytokines was mediated via the activation of MAPKs by inhibiting the phosphorylation of p38 and the extracellular signal‐regulated kinase (ERK). The MAPK pathway is a key intracellular signaling cascade in the immune response, involving three subgroups (ERKs, JNKs, and p38) in cell growth and death. Studies have indicated that natural products from plants that inhibit the p38 activity possess potent anti‐inflammatory properties.^[^ ^34^ ^]^ DL1 exhibited a weak effect on NF‐κB, whereas it reduced the phosphorylation of P‐IKKa/β (Figure 8, part B).

**Figure 9:**
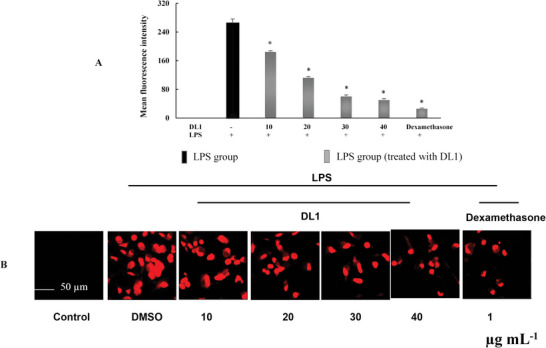
Detection of ROS via fluorescence intensity measurements. The fluorescence intensity of RAW 264.7 cells (A) and ROS identified as red fluorescence (B). *p < 0.05, compared to the LPS group.

Reactive oxygen species (ROS) have adverse effects on cell structure and functions. Excessive production of ROS can disrupt the redox balance of cells, leading to cell damage, thus accelerating the development and maintenance of inflammation, thereby affecting organs in the body and causing many diseases such as cancer and cardiovascular disease.^[^ ^35^ ^]^ Treatment with DL1 (30–40 µg mL^−1^) significantly reduced ROS release levels. DL1 exhibited an effectiveness similar to that of dexamethasone (1 µg mL^−1^, **Figure** **9**).

Previous studies have demonstrated that ROS mediates the activation of MAPK pathways. Therefore, ROS‐scavenging biomaterials prevent ROS accumulation by blocking MAPK activation.^[^ ^36^ ^]^

## 3 Conclusion

This is the first study to isolate and perform bioactivity studies on polysaccharides extracted from *H*. *ringens*. The isolated DL1 is a polysaccharide containing a xylose‐rich fraction (78.5% xylose) with highly esterified uronic acid moieties. DL1 belongs to the xylan group; it also contains 21.5% 4‐*O*‐methylglucuronic acid and is thus a 4‐*O*‐methylglucuronoxylan. DL1 protects bovine serum albumin from denaturation, with IC_50_ values of 0.35 mg mL^−1^. Furthermore, it has a similar activity to the non‐steroidal anti‐inflammatory drug diclofenac. DL1 strongly inhibited proinflammatory cytokine production (TNFα, IL‐6, and IL‐8) as well as the release of ROS. DL1 may inhibit cytokine release from LPS‐stimulated macrophages, mediated by the activation of MAPKs by downregulating p38 and ERK phosphorylation. The results of our study provide novel information on *H. ringens*, setting the basis for further studies while proving the effectiveness of this folk medicinal plant against swelling and inflammation.

## 4 Experimental Section

### Collection and Extraction of Polysaccharides

The seeds of *H. ringens* were collected from Can Tho City, Vietnam, in 2022. The samples (N°: VHB‐01.2022) were identified and stored at the Vietnam Academy of Sciences and Technology (VAST).

Polysaccharides from the *H. ringens* seeds were extracted with warm water upon continuous stirring.^[^ ^12^ ^]^ The seeds of *H. ringens* were soaked in water at 70 °C at a ratio of raw material to water of 1:20 (g mL^−1^). A highly viscous solution was formed around the seeds. The extract was filtered and the extraction process was repeated 6 times (1 h each time) until all soluble polysaccharides were extracted (the polysaccharides were determined using the phenol–sulfuric acid assay).^[^ ^37^ ^]^ The polysaccharides were precipitated with 70% ethanol, collected, and redissolved in warm water. Proteins were removed by adding 5% TCA. The polysaccharides were purified using a DEAE–cellulose 52 column (1.5 cm × 2 cm) and Sephadex G‐100 column chromatography (1.6 × 90 cm).^[^ ^3^ ^]^ The carbohydrate content of the fractions was analyzed using the phenol–sulfuric acid assay.^[^ ^37^ ^]^ The presence of proteins in the fractions was determined using the Lowry method.^[^ ^16^ ^]^

### Spectrophotometry and Spectroscopy

The purity of the polysaccharides was analyzed via UV‐Vis spectrophotometry following the method described by Li et al.^[^ ^15^ ^]^ The polysaccharide sample (100 µg mL^−1^) was completely dissolved in warm water and then scanned on a UV–Vis spectrophotometer (UV‐1650 (PC)S, Shimadzu Corporation, Japan) at wavelengths between 200 and 700 nm.

FT‐IR measurements were performed using an FT‐IR Spectrum Two (Perkin Elmer, USA). Dried samples of DL1 (2 mg) were mixed with KBr (10 mg) and compressed to a clear disc. The spectra were measured in the range of 4000 to 500 cm^−1^. DL1 was dissolved in D_2_O together with a few crystals of trimethylsilylpropanesulfonic acid as an internal standard and the solution was centrifuged before measurement. NMR spectra were recorded on a Bruker AVANCE 400 instrument at 60 °C using pulse sequences for ^1^H, ^13^C, DEPT135, COSY, HMBC, and HSQC/DEPT NMR.

### Molecular Mass Measurement

Molecular mass measurements were conducted using a JASCO SEC system equipped with a PU‐980 pump, PSS Suprema 3000 Å, and 300 Å columns (Polymer Standards Service GmbH, Mainz, Germany), as well as a RI‐930 refractive index (RI) detector (JASCO GmbH, Groß‐Umstadt, Germany). Measurements were performed at 30 °C and a 0.1 m NaNO_3_ aqueous solution was used as the eluent. The columns were calibrated using pullulan standards (Sigma‐Aldrich Chemie GmbH).

### Monosaccharide Composition and Linkage Analysis

The monosaccharide composition was evaluated by methanolysis and gas chromatography (GC) following a previously described method with modifications.^[^ ^38^, ^39^ ^]^

The samples were subjected to methanolysis in anhydrous methanol (MeOH) with 3 m hydrochloric acid (HCl) at 80 °C for 24 h. Trimethylsilylated (TMS) derivatives of the methyl glycosides obtained after methanolysis were analyzed on a Trace 1300 GC instrument (Thermo Scientific). Chromeleon Software v.6.80 (Dionex Corporation, Sunnyvale, CA, USA) was used for GC data analysis.

The glycosidic linkage types were determined via methylation followed by GC‐MS. The uronic acids in the polymers were reduced to neutral sugars prior to methylation. Carboxyl esters were reduced using sodium borodeuteride in an imidazole buffer to generate 6,6‐dideuteriosugars. The free uronic acids were activated using carbodiimide (*N*‐cyclohexyl‐*N*‐(2‐morpholinoethyl)‐carbodiimide methyl‐*p*‐toluenesulfonate) and reduced with sodium borodeuteride.^[^ ^40^ ^]^ After the reduction of the polymers, methylation was carried out according to the method described by Ciucanu.^[^ ^41^ ^]^ After the methylation procedure, hydrolysis of the glycosidic linkages and acetylation followed, as described by Kim and Carpita.^[^ ^40^ ^]^ The derived partially methylated alditol acetates were analyzed using a GC‐MS QP2010 (Shimadzu) with a Restek Rxi‐5 MS column (30 m, 0.25 mm i.d., 0.25 µm film thickness). The temperature of the injector and interface was 280 °C. The sample (1 µL) was injected at 80 °C and the temperature was increased to 140 °C at 10 °C min^−1^ °C, followed by an increase to 210 °C at 4 °C min^−1^, and a final increase to 310 °C (20 °C min^−1^) where it was kept for 4 min. Helium was used as the carrier gas. The spectra were analyzed using GC‐MS solution software v.2.10 (Shimadzu Corporation). The estimation of the relative amounts of each linkage type was related to the total mole percentage of their monosaccharide compositions determined after methanolysis and GC analysis, and the effective carbon response factors were considered for quantification.^[^ ^42^ ^]^

### In Vitro Anti‐Inflammatory Activity Evaluation

The anti‐inflammatory activity of DL1 against albumin denaturation was determined following a previously described method.^[^ ^43^ ^]^ BSA 0.5% (0.45 mL) was mixed with 50 µL of either the *H. ringens* polysaccharide, diclofenac (positive control), or distilled water (negative control) at concentrations between 0.1% and 1%. The mixtures were incubated for 20 min at 37 °C and then heated at 70 °C for 10 min, followed by cooling to room temperature. A phosphate buffer solution (pH 7.4, 2.5 mL) was added and the absorbance was measured at 660 nm.

(1) $$\begin{array}{c} inhibition\;of\;BSA\;denaturation\;\left(\%\right)=\frac{1-OD_{660\;nm}\left(sample\right)}{OD_{660\;nm}\left(negative\;control\right)}\times 100\% \end{array}$$

### Cell Viability Assay

RAW 264.7 macrophage cells were obtained from ATCC (American Type Culture Collection). Cells were seeded into 96‐well plates at a density of 5 × 10^4^ cells mL^−1^ in Dulbecco's modified eagle medium, 10% fetal bovine serum (FBS) at 37 °C, and CO_2_ (5%) for 24 h in a cell culture incubator. Polysaccharide solutions prepared in the culture medium at concentrations of 5, 10, 20, and 40 µg mL^−1^ were added to the cell culture wells and maintained at 37 °C with CO_2_ (5%) in an incubator for 24 h. The polysaccharide‐treated cells were incubated for 1 h with 10 µL of a CCK‐8 solution. The absorbance was measured at 450 nm using an ELISA reader (BioTek Synergy HT microplate reader, USA).

The viability was calculated as the percentage of the average OD value of the experimental sample compared to the average OD value of the positive control (treatment with H_2_O) according to Equation (2).

(2) $$Cell\;survival\;\left(\%\right)=\frac{OD_{450\;nm}\left(Sample\right)}{OD_{450\;nm}\;\left(Control\right)}\times 100\%$$

### Enzyme‐Linked Immunosorbent Assay and Western Blotting Analysis

RAW264.7 was a cell line widely used in anti‐inflammatory studies. The RAW264.7 macrophages were pretreated with the polysaccharide for 45 min. The cells were treated with LPS (lipopolysaccharide from gram‐negative bacteria) for 18 h, and the supernatant was collected to determine the concentration of cytokines using BD Bioscience ELISA kits (Mouse TNF ELISA Kit 555268, Mouse IL‐6 ELISA Kit 550950, Mouse IL‐8 ELISA Kit MBS261967, and Mouse Kit ELISA IL‐10 555252).^[^ ^44^ ^]^ The highest cytokine release content of the macrophage cell group induced with LPS without the polysaccharide sample was calculated as 100%, and the cytokine content of the LPS group with the added polysaccharides was compared with the LPS‐induced group (*A*%). The inhibition of cytokine release of the polysaccharide (% relative activity) was calculated as 100% − *A*%.

The effects of DL1 on the MAPK signaling pathways of RAW264.7 cells were determined by western blot assay as previously described.^[^ ^45^ ^]^ Antibodies against phospho‐(Thr202/Tyr204)‐ERK1/2 (9101), phospho‐(Thr180/Tyr182)‐p38 (9211), phospho‐IKKα/β (Ser176/180) (2697), IkBα (4812), and α‐actin (19245) were obtained from Cell Signaling (Beverly, MA). The RAW 264.7 cells were treated with the polysaccharide (10, 20, 30, and 40 µg mL^−1^) in the presence or absence of 1 µg mL^−1^ LPS. The membranes were detected using a chemiluminescence (ECL) reagent (Amersham‐Pharmacia, Little Chalfont, UK).

### Assessment of ROS Inhibition

After incubating with or without DL1 for 45 min, the macrophages were treated with LPS and were incubated with dihydroethidium (Merck, Germany) for 15 min at 37 °C under the exclusion of light in a 5% CO_2_ atmosphere. The cells were observed using laser scanning confocal microscopy (LSM 510; Zeiss, Oberkochen, Germany) to determine the different concentrations of ROS in the treated samples. The fluorescence was measured at an excitation wavelength of 488 nm and an emission wavelength of 515–540 nm and the mean relative fluorescence intensity was measured using a Carl Zeiss vision system (Germany).^[^ ^46^ ^]^

### Statistical Analysis

Statistical analysis was expressed as ±SD and analyzed via one way ANOVA and Dunnett's test for multiple comparisons. Values were considered significantly different at p* < 0.05. The IC_50_ values were analyzed using GraphPad Prism 8 (version 8.0.2).

## Conflict of Interest

The authors declare no conflict of interest.

## Author Contributions

V.H.B. dealt with the project administration, data curation, formal analysis, investigation, and writing original draft. T.C.T. dealt with the investigation and formal analysis. A.K. dealt with the investigation, formal analysis, writing the review, and editing. T.H. dealt with the data curation, writing the review, and editing. Y.P.F. dealt with the investigation and analysis. K.T.I. dealt with data curation, formal analysis, writing review, and editing. L.V.T. dealt with the investigations. B.S.P. dealt with the formal analysis, writing review, and editing. M.G. dealt with the investigations.

## Supporting information



Supporting Information

## Data Availability

The data that support the findings of this study are available from the corresponding author upon reasonable request.;
