# Supplementary material for: 4‐O‐Methylglucuronoxylan from Hygrophila Ringens var. Ringens Seeds: Chemical Composition and Anti‐Inflammatory Activity
Source: Macromol Biosci. 2025 Jan 13;25(4):2400434. doi: 10.1002/mabi.202400434 (PMC11995837; doi:10.1002/mabi.202400434)
Supplement: Supplementary file 1 — Supporting Information [file MABI-25-2400434-s001.docx]

Supporting Information

**4-*O*-Methylglucuronoxylan from *Hygrophila ringens* var. *ringens* Seeds: Chemical Composition and Anti-inflammatory Activity**

*Vo Hoai Bac*, Tat Cuong Trinh, Andreas Koschella, Thomas Heinze^*^, Yu Ping Fu, Kari Tvete Inngjerdingen_,_ Le Van Truong, Berit Smestad Paulsen, Martin Gericke*

Author(s), and Corresponding Author(s)*

Vo Hoai Bac*

Institute of Biotechnology, Vietnam Academy of Science and Technology, 18 Hoang Quoc Viet, 100000 Hanoi, Vietnam

Graduate University of Science and Technology, Vietnam Academy of Science and Technology, 18 Hoang Quoc Viet, 100000 Hanoi, Vietnam

Email: [*vhbac@ibt.ac.vn*](mailto:vhbac@ibt.ac.vn) *or* [*vhoai1812@gmail.com*](mailto:vhoai1812@gmail.com)

Thomas Heinze*

Friedrich Schiller University Jena, Institute for Organic Chemistry and Macromolecular Chemistry, Center of Excellence for Polysaccharide Research, Humboldtstraße, D-07743 Jena, Germany

Email: [*thomas.heinze@uni-jena.de*](mailto:thomas.heinze@uni-jena.de)


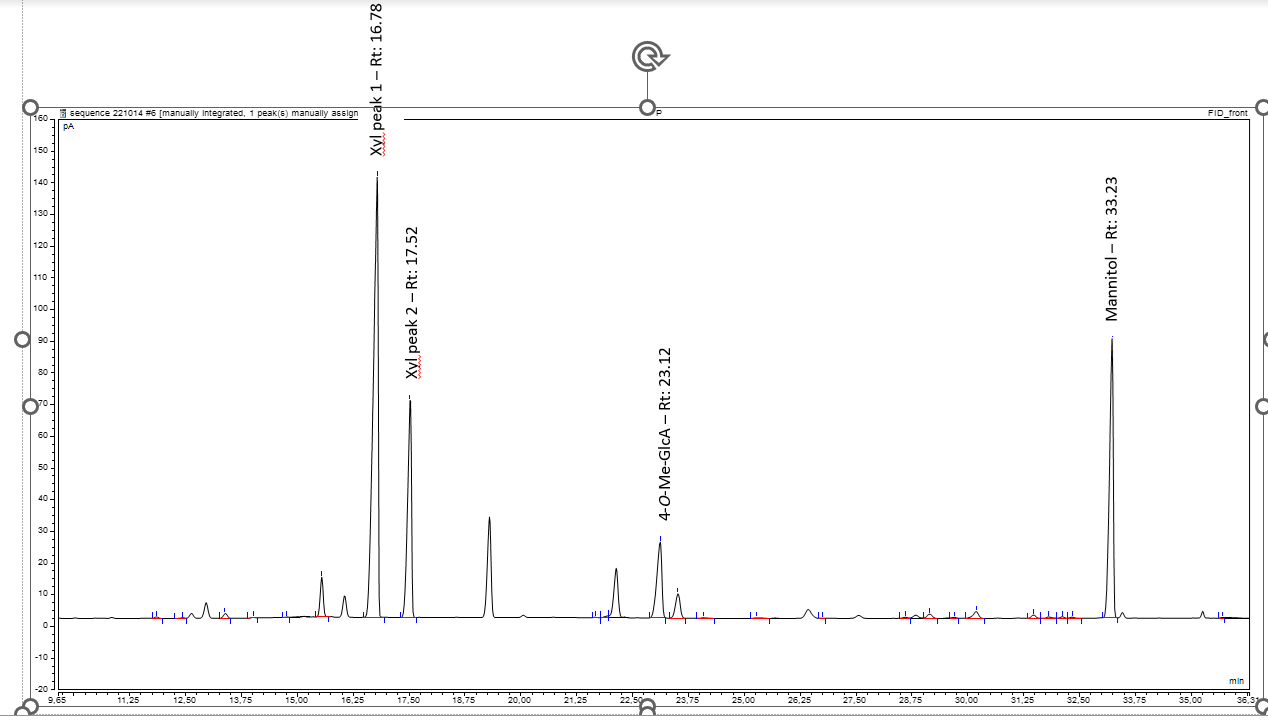


Figure S1. The GC chromatogram after methanolysis of DL1


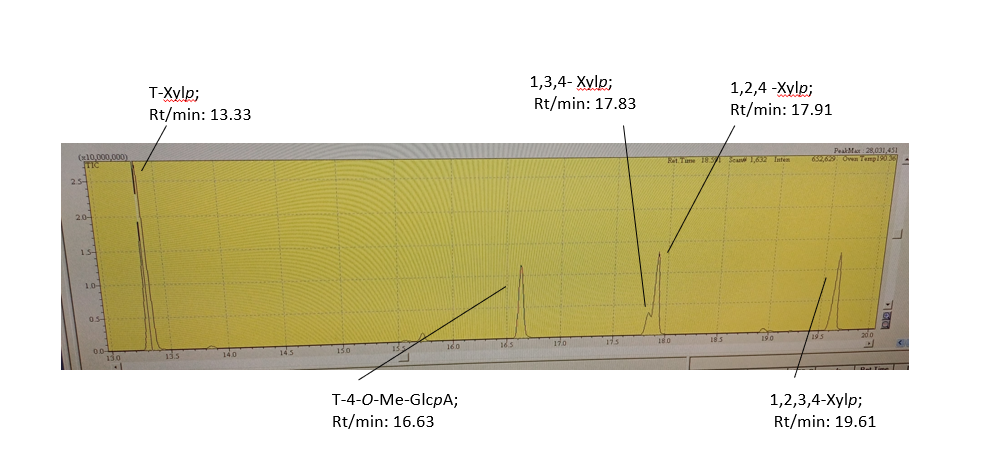


**Figure S2.** GC chromatogram of partially methylated alditol acetates of **DL1** after derivatization for linkage analysis.


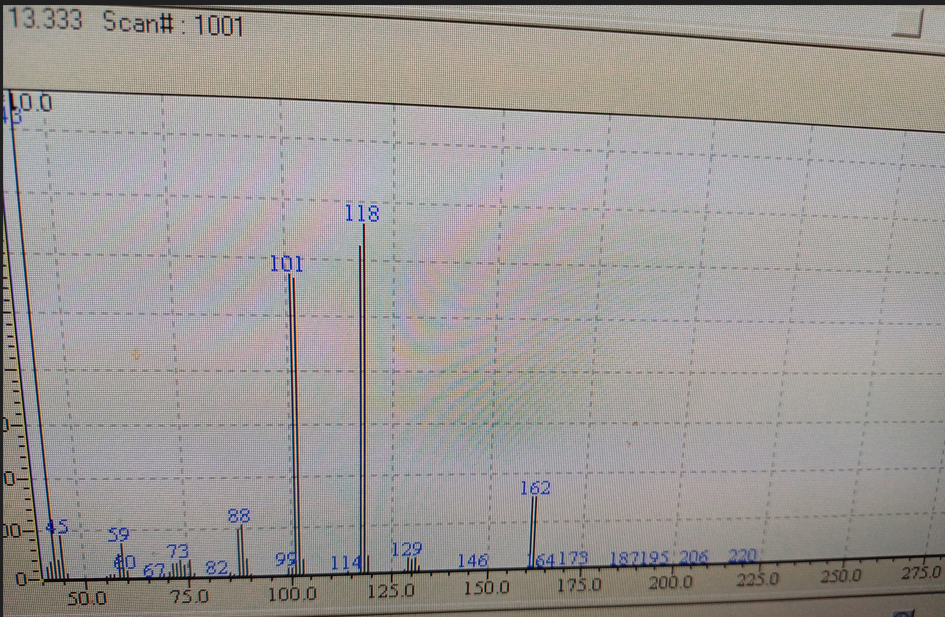


T-Xyl*p*;

Rt/min: 13.33


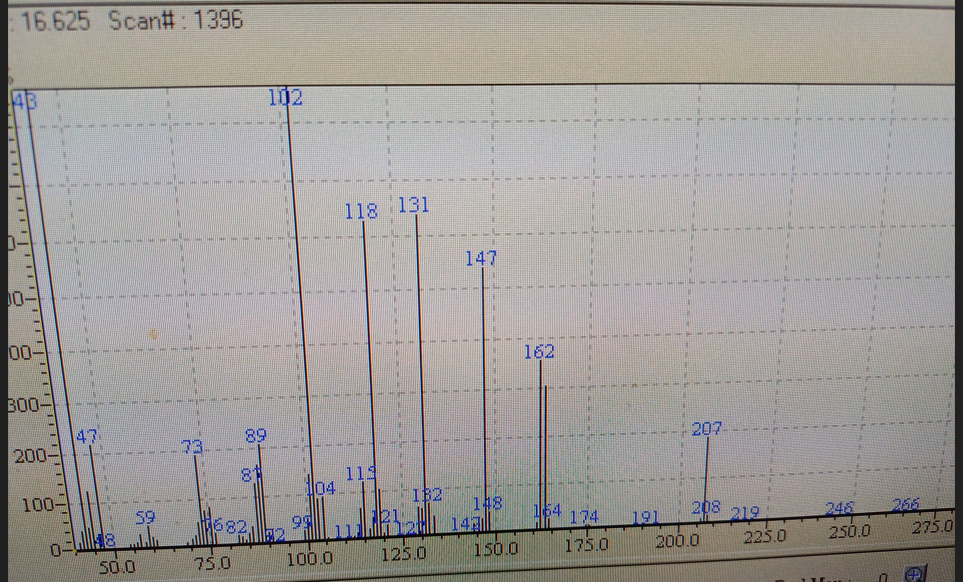


T-4-*O*-Me-Glc*p*A;

Rt/min: 16.63

**
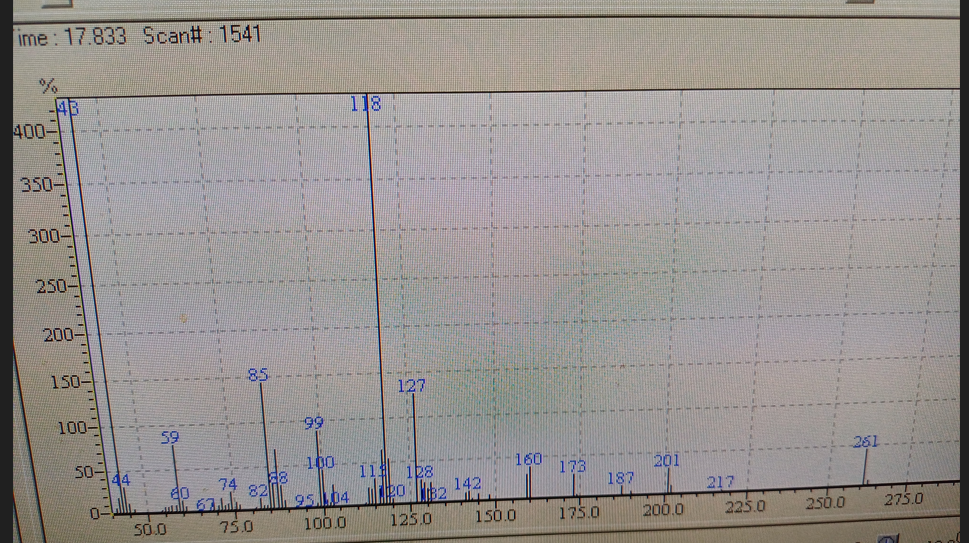
**

1,3,4- Xyl*p*;

Rt/min: 17.83

**
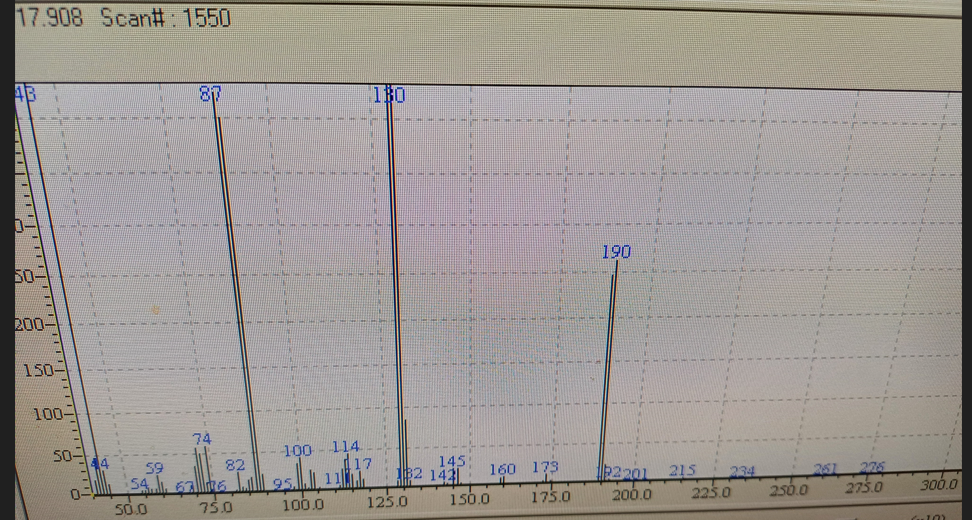
**

1,2,4 -Xyl*p*;

Rt/min: 17.91


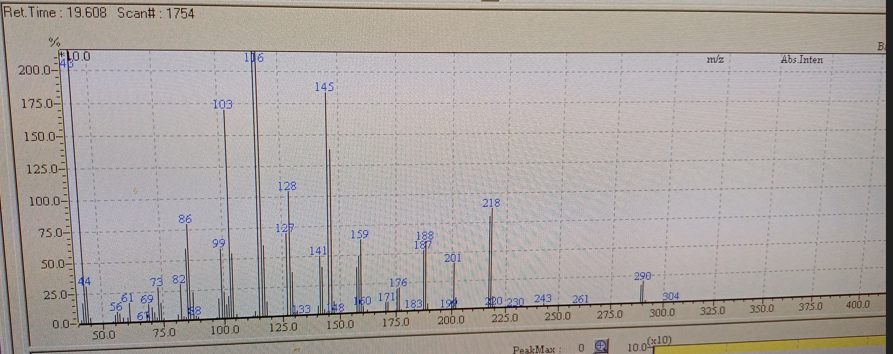


1,2,3,4-Xyl*p*;

Rt/min: 19.61

**Figure S3.** MS spectra of partially methylated alditol acetates of **DL1** after derivatization for linkage analysis.


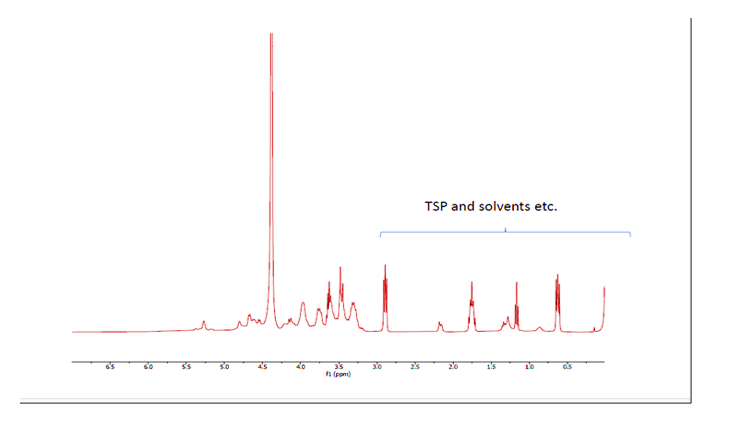


**A**


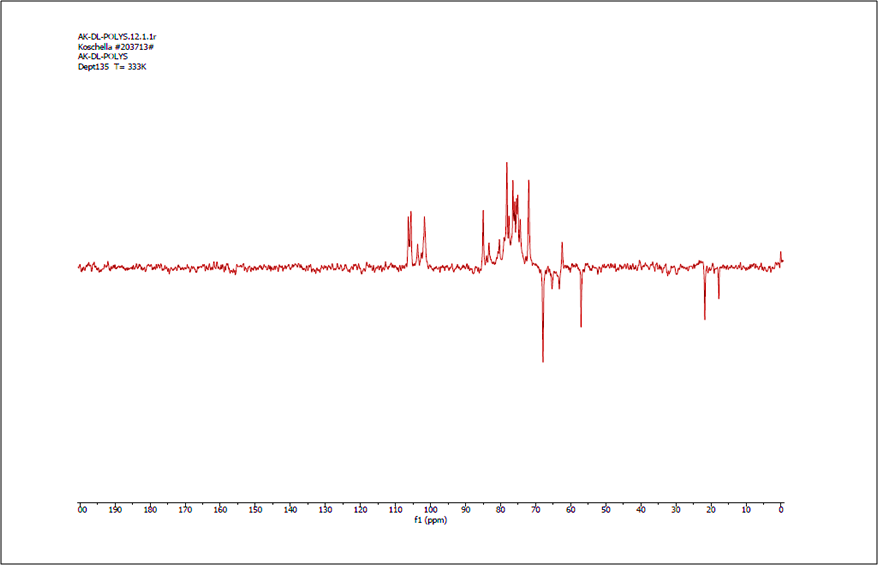


**B**


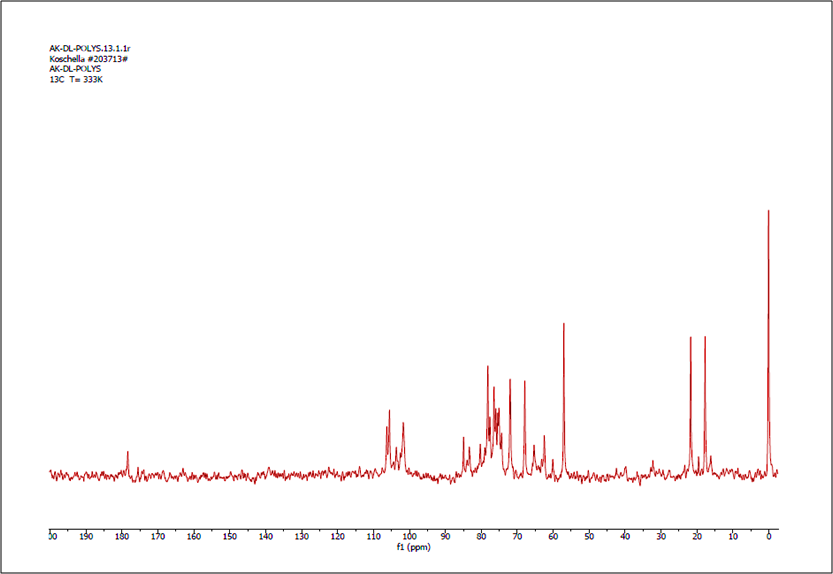


**C**


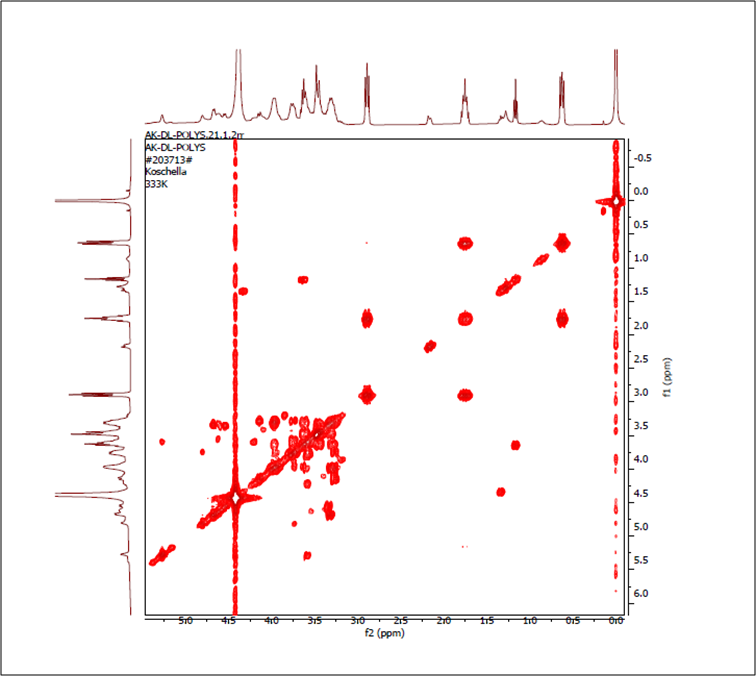


**D**

**
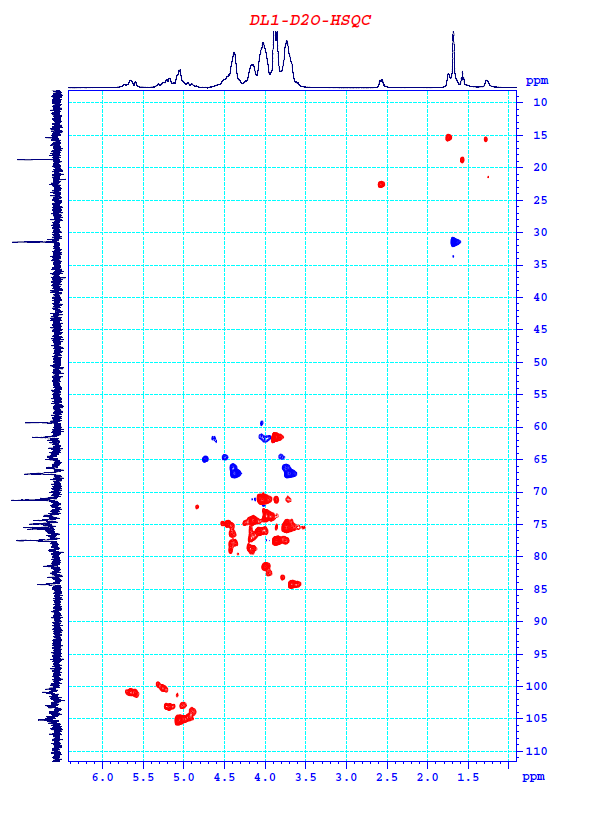
**

**E**

**
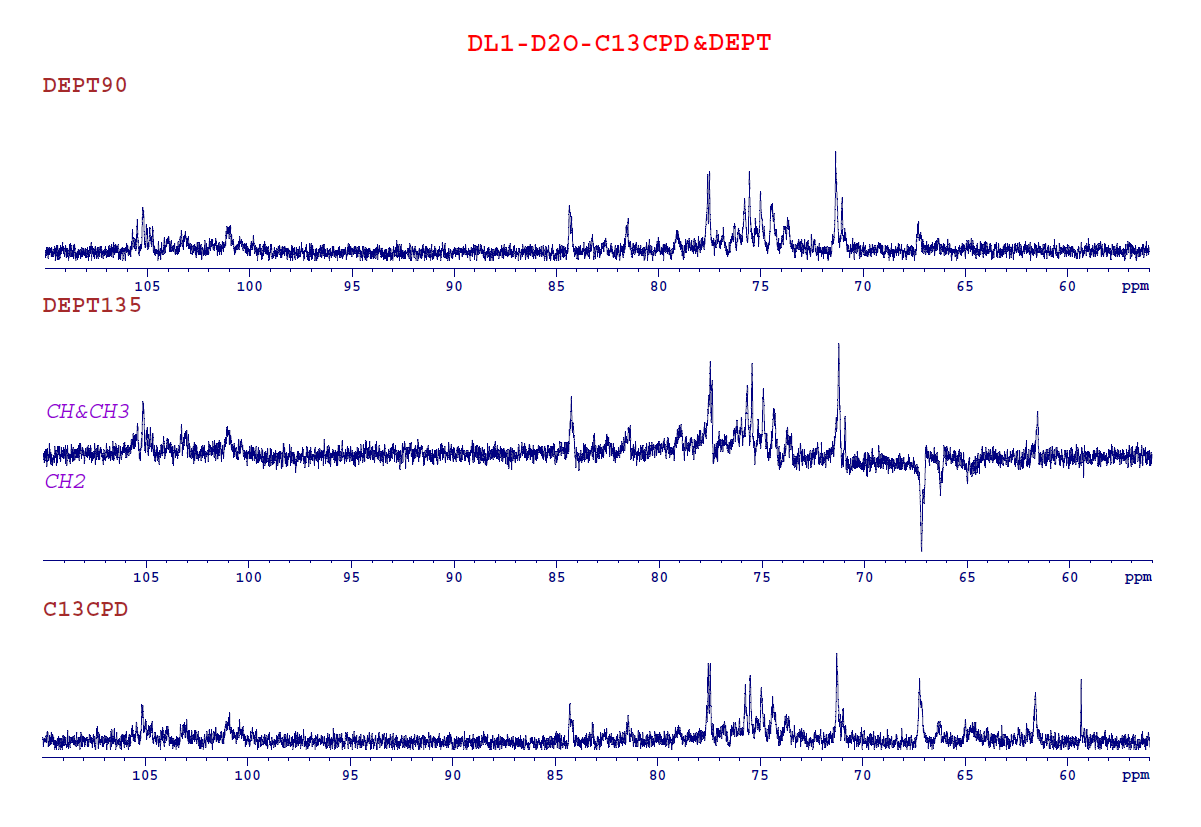
**

**F**

**
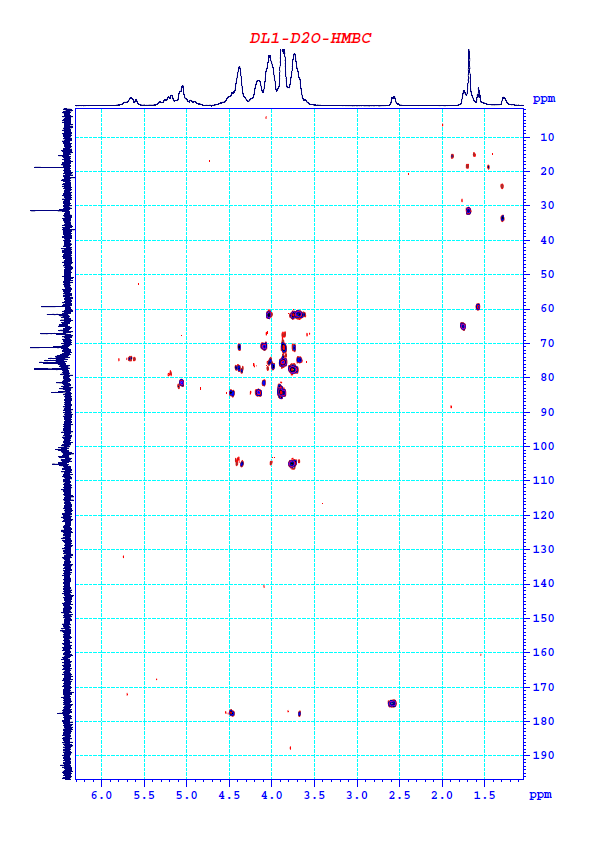
**

**G**

**Figure S4.** ^1^H- (A), ^13^C- (B, C), ROESY- (D), HSQC- (E), DEPT- (F) and HMBC- NMR spectra (G) of sample **DL1** recorded in D_2_O.

**
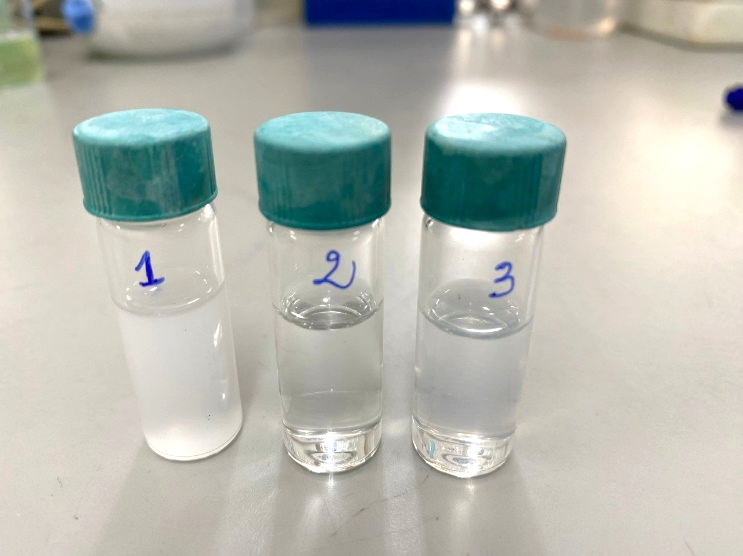
**

**Figure S5.** BSA without (1), with Diclofenac (1 mg mL^-1^, 2) and **DL1** (1 mg mL^-1^, 3) after thermal treatment.

**
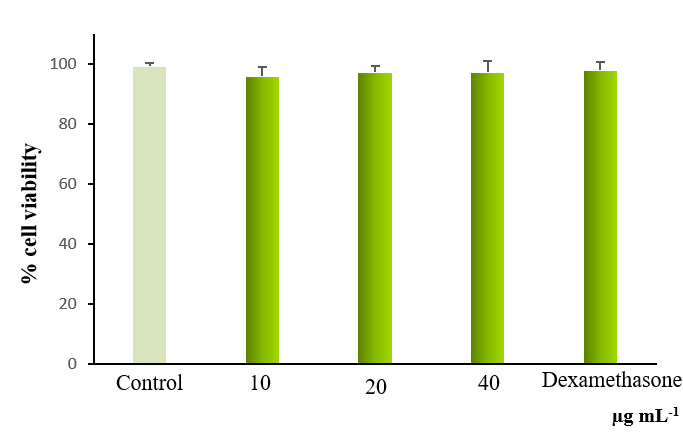
**

**Figure S6.** Effect of **DL1** on RAW 264.7 macrophage proliferation**.** : Control group (treatment with H_2_O); :group were treated with **DL1**.
